# Exploiting the Opportunity to Use Plant-Derived Nanoparticles as Delivery Vehicles

**DOI:** 10.3390/plants12061207

**Published:** 2023-03-07

**Authors:** Vincenza Tinnirello, Nima Rabienezhad Ganji, Carine De Marcos Lousa, Riccardo Alessandro, Stefania Raimondo

**Affiliations:** 1 Department of Biomedicine, Neuroscience and Advanced Diagnostics (Bi.N.D), Section of Biology and Genetics, University of Palermo, 90133 Palermo, Italy; 2 Biomedical Sciences, School of Health, Leeds Beckett University, Leeds LS1 3HE, UK; 3 Centre for Plant Sciences, University of Leeds, Leeds LS1 3HE, UK

**Keywords:** plant-derived nanoparticles (PDNPs), drug delivery, co-incubation, sonication, microRNA

## Abstract

The scientific community has become increasingly interested in plant-derived nanoparticles (PDNPs) over the past ten years. Given that they possess all the benefits of a drug carrier, including non-toxicity, low immunogenicity, and a lipid bilayer that protects its content, PDNPs are a viable model for the design of innovative delivery systems. In this review, a summary of the prerequisites for mammalian extracellular vesicles to serve as delivery vehicles will be given. After that, we will concentrate on providing a thorough overview of the studies investigating the interactions of plant-derived nanoparticles with mammalian systems as well as the loading strategies for encapsulating therapeutic molecules. Finally, the existing challenges in establishing PDNPs as reliable biological delivery systems will be emphasized.

## 1. Introduction

Natural nanoparticles are nanosized vesicles secreted by virtually all organisms. Mammalian nanoparticles have been studied extensively and are comprised of numerous types of particles that are classed depending on their origin, size, and density. The published MISEV guidance attempts to give clarity on this classification as well as isolation and purification recommendations [1]. Thanks to the constant and rapid evolution of knowledge, this guidance is constantly updated [2] to give an accurate and timely state of knowledge in the field.

Despite both plant and mammalian nanoparticles having been observed at similar times (the late 1960s and early 1970s, respectively), the field of plant nanoparticles remained relatively unexplored until they were shown to support interspecies communication with mammalian cells [3] and with plant pathogens [4]. This discovery has promoted in recent years the fast development of plant nanoparticle research and attracted much interest in their potential as a drug delivery vehicle, similar to mammalian nanoparticles.

Plant-derived nanoparticles (PDNPs) can be classified in two main groups: plant extracellular vesicles (EVs), isolated from an extracellular medium such as the apoplast, and plant-derived nanovesicles (PDNVs) isolated from a whole plant extraction such as juice and containing plant EVs as well as other nanovesicles [5,6]. Whereas PDNVs can originate from a variety of membranes, the origin of EVs is more defined. Proposed EV classes include three subgroups: exosomes (originating from MVBs), microsomes (originating from the plasma membrane), and EXPO vesicles [7]. Unlike for mammalian nanoparticles, where numerous protein markers have been identified, PDNP markers are scarce due to the relative variety of plant species studied, which hinders the search for a universal marker due to cross-reactivity issues. However, two main proteins, suggested to be specific for different EV populations, have been established as genuine *A. thaliana* EV markers: TET8, the homologue of mammalian CD63 [8] found in exosomes and PEN1, a syntaxin protein [9].

Despite the lack of specific markers, one of the most important features of PDNPs is their complex content, consisting of DNA, RNA, proteins, lipids, and biologically active components. The precise nature of the PDNP’s content is difficult to establish, as it will depend on the plant species and the nanoparticle extraction process. A number of biologically active molecules, however, have been found to be playing important roles, not only in normal plant development, but also in cross-kingdom interactions between plants and their pathogens as well as between PDNPs and mammalian cells. In particular, various groups have demonstrated a transfer of biological material, more specifically of sRNAs, from PDNPs to mammalian cells (see below). The question arose as to whether PDNPs are capable of delivering therapeutic molecules and acting as delivery vehicles. Growing evidence seems to show that PDNPs might represent the next generation of drug delivery vehicles due to various advantages: not only is their ability to deliver biologically active/therapeutic molecules documented, but their ability to cross mammalian biological barriers, their high stability, and low production costs are all qualities that are attractive for biopharmaceutical purposes.

Here, we will give a summary of the requirements for mammalian EVs to act as delivery vehicles as well as the challenges faced by the field. We will compare this with the current knowledge on PDNPs acting as potential delivery vehicles. We will focus on giving an extensive overview of the current field of plant-derived nanoparticle research on the cross-interaction with mammalian systems and the main loading methods for encapsulating therapeutic molecules. Finally, we will discuss the current challenges to establishing PDNPs as genuine delivery vehicles for biomedicine.

## 2. Mammalian Extracellular Vesicles as Delivery Vehicles

So far, in the field of nanomedicine, attention has mainly focused on the study of animal-derived extracellular vesicles (EVs). Indeed, ever since their discovery, it became clear that their natural function in intercellular communication could be exploited for the development of new drug delivery systems. In addition to this natural inclination, they possess a long list of other qualities that make them more than just cellular structures.

For example, they are stable in the circulation due to their negatively charged surface and poorly immunogenic due to their ability to avoid the mononuclear phagocytic system because of the presence of the surface protein CD47. They have no toxicity and are also intrinsically capable of crossing physical biological barriers, including the blood–brain barrier [10,11].

In addition to the substances they inherently transport and deliver, several research groups have successfully loaded therapeutic substances such as proteins, nucleic acids, or small molecules into EVs. To date, two types of approaches have been used for this purpose: (1) In the first approach, which includes so-called exogenous methods, substances are loaded inside isolated EVs; (2) In the second, the loading of substances inside EVs occurs during their biogenesis, by engineering the cells prior to EV isolation [12]. The first class of loading approaches can be divided into passive and active methods. Passive loading mainly refers to the co-incubation of EVs with molecules of interest, which are often, but not always, hydrophobic substances capable of crossing the vesicle membrane by simple diffusion. Several reports have shown that substances such as curcumin [13], or anticancer drugs, e.g., doxorubicin or paclitaxel [14,15,16], or even therapeutic nucleic acids, such us miRNA [16,17], can be successfully loaded inside extracellular vesicles by simple incubation. Active loading, on the other hand, involves a momentary permeabilization of the EV membrane. This permeabilization can occur by physical methods such as high-voltage electrical impulses or sonic waves that cause ruptures in the EV membrane, as occurs in electroporation and sonication, respectively. Although these methods have limitations such as the formation of fused vesicles or structural distortions of the membrane, many research groups have used them with excellent results to load a wide variety of substances including anticancer drugs [18,19,20,21], nucleic acids of various types such as DNA, RNA, and small non-coding RNA [10,22,23,24,25,26], and nanomaterials [27,28]. Alternatively, loading can occur through the addition of surfactants such as saponins that remove cholesterol molecules, leaving pores in the membrane through which molecules of interest can enter. To date, the technique is described in works where several loading techniques are compared with the aim of establishing the best method for loading capacity and efficiency [29,30,31,32]. Other recognized, though less used, loading methods are extrusion, freeze-thaw cycles, and dialysis. As with the use of saponins, the first two are less explored and less applied in comparative work [29,31,33]. Loading can be carried out through hypotonic dialysis, which is based on osmosis. The EVs are dispersed in a hypotonic solution, which causes them to swell, resulting in the formation of pores allowing the drug to be loaded. The loaded vesicles are then dispersed in an isotonic solution to restore the integrity of the EVs [34,35]. With a similar system, it is also possible to create a pH gradient that favors the loading of miRNA and siRNA into the vesicles [36]. As already mentioned, there are so-called endogenous loading techniques. In these techniques, the cargo is loaded prior to isolation of the extracellular vesicles. This can be done by incubating drugs and/or nanomaterials with EV-secreting cells. Small molecule drugs such as the already discussed paclitaxel, curcumin, or doxorubicin can freely pass through the lipid bilayer of cells and there be further packaged into intraluminal vesicles subsequently secreted as exosomes [37,38,39,40]. The most used methods for the loading of endogenous compounds in EVs are based on modifications of EV-producing cells. These approaches involve the use of specific transfection reagents or plasmids that induce cells to ectopically express the nucleic acids [41,42,43,44,45], proteins, and peptides of interest [46,47,48,49], which are then sorted into EVs. The above-mentioned works are only a part of those currently in the literature. They show that mammalian extracellular vesicles have always received much attention, being the subject of innumerable studies and applications. However, it must be considered that their use as a drug delivery system presents critical issues that often do not allow their clinical use, in particular the low production scale together with the high production costs due to the sophisticated technologies required. This problem could be overcome by using extracellular vesicles derived from plants. While they possess all the safety features in common with mammalian-derived EVs, they present the possibility of being produced in large quantities and at low cost.

## 3. Plant-Derived Nanoparticles as Delivery Vehicles

Plant-based synthetic nanoparticles have already been used for therapeutic purposes; for example, zinc oxide nanoparticles were used as anticancer and antibacterial strategies [50]. Those produced from the leaf extract of Cassia fistula and Melia azedarach exhibited impressive antimicrobial action against clinical pathogens in comparison to conventional drugs [51]. It has long been debated whether plant matrices could be also used to develop controlled delivery systems, representing a suitable alternative to inorganic nanoparticles. For instance, the production of hydrogels to improve drug solubility has made significant use of cellulose [52]. Plant protein-based nanoparticles have been generated. Zein, derived from cereals, was able to deliver anticancer drugs like doxorubicin [53] and 5-fluorouracil 21094232, whereas gliadin, derived from gluten, served to produce drug release control systems for B-carotene [54] and vitamin E [55]. Despite the promising results, the use of these vectors required a synthetic approach.

Possessing all the advantageous properties for a drug delivery vehicle, including safety, non-toxicity, low immunogenicity, plant-derived nanoparticles (PDNPs) represent a promising model for the development of drug delivery systems, especially due to their physiological origin. Furthermore, different studies have shown that they are stable and resistant in the stomach; in fact, after oral gavage, PDNPs have been found in intestinal stem cells, liver, colon, and dendritic cells, and in mice intestinal macrophages [56,57,58,59,60]. Based on these very promising features, we will discuss below what is already known about the use of PDNPs in the field of drug delivery.

### 3.1. Non-Modified PDNPs: Endogenous Content and Biological Functions

PDNPs already possess their endogenous content: a load of lipids, proteins, mRNAs, miRNAs, and other functional substances are normally enclosed within these small vesicles. Although it is difficult to correlate a biological property with a single compound, some researchers have succeeded in the association. Deng et al. highlighted the ability of broccoli-derived nanoparticles (BDNs) in regulating intestinal immune homeostasis through adenosine monophosphate kinase (AMPK)-mediated activation in dendritic cells (DCs) and in preventing colitis in vivo in three colitis models in mice [60]. Interestingly, these effects are related to a substance in which BDNs are enriched: sulforaphane (SFN).

Using knockout and knock-in strategies, SFN-depleted nanoparticles (SFN knockout SFN−/−), and SFN knock-in nanoparticles (SFN+/+) were generated. In mice, adaptive transfer of SFN+/+ pre-treated DCs protected the animals from colitis compared to those that had received SFN−/− pre-treated DCs. In these animals, there was less tissue damage, and less reduction in colonic length as well as a lower level of the pro-inflammatory cytokines IFNγ and TNFα compared to mice that received the SFN−/− treated DCs [60]. In a 2020 paper, Song et al. demonstrated that a surface protein present in garlic-derived nanovesicles (GDVs), lectin II, plays an important role in the cellular internalization of GDVs, a fundamental mechanism for GDVs to exert their anti-inflammatory effects [61].

Zhuang et al. demonstrated that ginger-derived nanoparticles (GDN) have a protective role against alcohol-induced liver damage. This property depends on the activation of erythroid nuclear factor 2-related (Nrf2), which induces the expression of detoxifying/antioxidant genes, thus inhibiting the production of reactive oxygen species (ROS). In this work, using the KO and knock-in strategy, they correlated the observed effects to the presence of shogaol, which is naturally present in GDNs and which plays a role in the induction of Nrf2 nuclear translocation in target hepatocytes through the activation of the TLR4/TRIF pathway [58].

The biological properties of GDVs, correlated to the presence of 6-gingerol and 6-shogaol, were also highlighted by Zhang et al. in a study on the prevention and treatment of inflammatory bowel disease and tumor caused by colitis [62]. The group isolated and identified three nanoparticle populations with similar lipid and protein profiles, but different levels of 6-gingerol and 6-shogaol. The results revealed that the administration to mice with DSS-induced colitis of GDVs with the higher content of the two compounds reduced acute inflammation compared to the other groups of animals treated with the other populations of GDVs. In addition, to emphasize the molecular mechanism induced by orally administered GDVs, they report the reduction in the expression of pro-inflammatory cytokines (TNF- a, IL-6, and IL-1b) and the increase in the expression of anti-inflammatory cytokines (IL-10 and IL-22), suggesting that they block factors that injure the gut and promote those that repair it [62]. Similarly, Teng et al. published a study revealing how small RNAs, and miRNAs contained in GDVs, modulate the composition of the gut microbiota. In this study, they show that GDV microRNA content targets specific Lactobacillus rhamnosus (LGG) genes, whereas metabolites released by LGG after GDV treatment control the growth of other gut bacteria. Among the identified miRNAs, an important role is played by miR-7267-3p, which, by interfering with the expression of the ycnE gene, regulates the level of IL-22, a cytokine crucial for the maintenance of the intestinal barrier function. Additionally, miR-167a, contained in GDV, inhibited transcription of a Pili gene, SpaC in Lactobacillaceae, thus decreasing bacterial mobilization from the gut to other organs [63]. GDV-transported miRNAs were also studied in a paper by Teng et al. on lung inflammation caused by SARS-CoV-2 [64]. In this study, they reported that aly-miR396a-5p, contained and transported by GDVs, prevents the inflammatory response caused by the injection of EVs from lung epithelial cells, through specific inhibition of viral Nsp12 gene expression [64].

Vesicle-like nanoparticles with relevant biological properties were also isolated from honey (H-VLNs) by Chen et al. [65]. H-VLNs analysis revealed the presence of several plant transmembrane proteins as well as of lipids and small RNAs. This is not surprising, considering that honey is derived from the processing of flower nectar by bees and thus carries some plant biological information in its H-VLNs.

The study firstly demonstrated the ability of H-VLNs to significantly inhibit the activity of the NLRP3 inflammasome by preventing the formation of the inflammasome complex. In addition, administration of these nanoparticles in a mouse experimental model of acute liver injury reduced inflammation and hepatic damage. Finally, miR4057, contained in H-VLNs, is considered responsible for the observed effects as a potent suppressor of Casp1 self-cleavage, a key event in the activation of the NLRP3 inflammasome [65]. These results demonstrate how PDNPs are already intrinsically important vehicles/transporters of valuable substances that exert relevant biological properties at the sites of interest. This has certainly attracted the interest of the scientific community, which has used PDNPs as a basis for building safe and effective nano-drugs.

### 3.2. PDNPs’ Loading Strategies: Types of Cargo, Membrane Modifications and Functional Effects

Potential therapeutic cargoes, which include RNA, proteins, and drugs, can be loaded into PDNPs and different strategies can be followed for this purpose. Although not many loading techniques have been applied with PDNPs, we know from experience with cell-derived exosomes that it is possible to load exogenous molecules employing passive and active loading techniques. Some of the most widely used are co-incubation, sonication, electroporation, and extrusion [12,13,19,23,31,66]. Due to the lipidic nature of the PDEV membrane, various hydrophobic or amphiphilic drug compounds can be passively loaded into exosomes by co-incubation. According to this technique, it is sufficient to incubate the PDNPs together with the molecule of interest at a given temperature. Loading will be driven by simple diffusion and lipophilic interactions. Using this system, Zeng et al. succeeded in encapsulating indocyanine green (ICG) within nanovesicles isolated from aloe gel (gADNVs) [67]. Despite being a recognized drug, there are some limitations to the use of free ICG. It is thermolabile and prone to binding to plasma proteins in the blood. Indocyanine green (ICG) loaded into gADNVs (ICG/gADNV) showed great stability both in the heating system and in serum, even after 30 days of gADNV storage. Furthermore, ICG/gADNVs stored for 30 days could still be successfully internalized by melanoma cells, thus inhibiting their growth, surpassing the effects of free ICG and ICG liposomes [67]. The same loading system was used by Xiao et al., who developed a drug delivery system based on lemon-derived extracellular vesicles loaded with doxorubicin and functionally modifying the surface of EVs with heparin-cRGD (HRED). HREDs were efficiently internalized by DOX-resistant ovarian cancer cells via caveolin-mediated endocytosis, exhibiting excellent cellular uptake and anti-proliferative properties, thus efficiently overcoming multidrug resistance [68]. A short incubation, without special reagents or electroporation, was sufficient for Umezu et al. to encapsulate miRNAs in Acerola exosome-like nanoparticles (AELNs). This study examined the possibility of developing a drug delivery system involving the oral administration of AELNs. It was found that administration of a mixture of AELNs/miRNAs into cells causes downregulation of the miRNA target gene. Furthermore, in an in vivo study, orally administered AELNs induced suppression of the target gene in the small intestine and liver, indicating the ability of AELNs to protect the encapsulated cargo from RNase and acid and base treatments and thus their potential use as delivery system [69]. Wang et al. used co-incubation in the presence of carbodiimide to conjugate grapefruit nanovesicles (GNVs) with methotrexate (MTX), an immunosuppressive and anti-inflammatory agent, to counteract DSS-induced colitis in mouse models. They observed that oral administration of the conjugates reduced not only colitis-related symptoms and colon damage induced by DSS, but also the gene and protein expression of pro-inflammatory cytokines, such as TNF-α, IL-1β, and IL-6, in intestinal macrophages in treated mice compared to untreated control groups or those treated only with free MTX. This demonstrated that MTX delivery within GNVs not only improved the anti-inflammatory effects of the drug, but also reduced its adverse effects [59]. In contrast, hydrophilic drugs and macromolecules require transient permeabilization of the vesicle membrane to be encapsulated. This is achieved by applying techniques such as electroporation or sonication [29]. To date, electroporation has found extensive applications in the context of extracellular vesicles, but has never been used in the field of plant extracellular vesicles. The technique makes use of the administration of short high-voltage pulses that can induce a transient and reversible rupture of the membrane. It is possible to exploit this rupture to load the molecules of interest inside the vesicles (reviewed in [70]). Although in most cases work using electroporation shows good loading efficiency (about 20–25% [10,18]), the technique has limitations: for example, one of the most common disadvantages is the aggregation or fusion of vesicles because of the electrical pulses [66,71,72]. Like electroporation, another widely used technique for loading extracellular vesicles of various origins is sonication. In this case, the momentary membrane permeabilization occurs by ultrasonic waves that damage the integrity of the membrane and facilitate the capture of drugs or other substances of interest [12,20,73,74]. There are not many studies in which sonication is used for loading in PDNPs. In this regard, Garaeva et al. succeeded in loading grapefruit extracellular vesicles with the anti-apoptotic protein HSP-70 by combining passive penetration of the protein with sonication [75]. Sonication can also potentially cause structural distortion of the membranes, resulting in the formation of fused vesicles. However, no significant changes in the morphology and size distribution of sonication-loaded GF-EVs were evidenced. Furthermore, using in vitro models, they demonstrated that the uptake of either fluorescently labelled and GF-EV-loaded HSP70-AF647 or BSA-AF647 proteins by human cells is significantly more efficient than that of free proteins. Furthermore, by exploiting the potent anti-apoptotic capabilities of HSP70, they demonstrated how, when enclosed within grapefruit vesicles, it induced increased resistance of human DLD1 tumor cells to etoposide, suggesting that the chaperone maintained its cytoprotective activity by being incorporated into the vesicles [75]. Sonication was also the method chosen by Yang et al. to load the drug 5-fluorouracil (5-FU) into bitter melon-derived extracellular vesicles (BMEVs) for the treatment of oral squamous cell carcinoma (OSCC) [76]. The results showed that BMEVs reduce the expression of NLRP3, which reduced the chemical resistance of OSCC cells to 5-FU, and BMEVs can suppress OSCC growth and induce apoptosis by activating ROS [76]. As with mammalian-derived extracellular vesicles, it is very common to use transfection techniques for loading nucleic acids and, in particular, miRNAs into PDNPs. In the study conducted by Lorenadel Pozo-Acebo, the potential of carrying exogenous miRNA by broccoli vesicles was investigated [77]. EVs were isolated from broccoli flower heads and then five groups of RNAs were extracted from these EVs, such as ath-miR159a, ath-miR159b-3p, ath-miR166b-3p, ath-miR319a, and ath-miR403-3p mimics. To assess if broccoli-isolated EVs could be loaded with exogenous miRNAs with potential therapeutic capacity, these were transfected (via lipofection) with the five previously selected miRNA candidates (enriched EVs). In this study, to understand the function of miRNA-loaded broccoli EVs, the complex was incubated with Caco-2 cells and the evidence showed that the cell viability decreased after the treatment. It was also found that EVs derived from broccoli can protect miRNA against RNase and gastrointestinal digestion [77]. You et al. also used transfection agents to encapsulate miR-184 within cabbage exosome-like nanovesicles and were able to deliver it to colon cancer cells [78]. Indeed, 72 h after administration to the cells, miRNA levels were measured by real-time PCR, and a 246,000-fold increase in miRNA levels was observed in the treated cells compared to the control. In the same study, the research team also investigated the possibility of encapsulating doxorubicin in nanoparticles derived from cabbage by simple incubation. The vesicles containing doxorubicin effectively suppressed the proliferation of colon cancer cells and cell viability was dramatically reduced after treatment of treated cells compared to controls (untreated cells and cells treated with free doxorubicin). These data suggested that doxorubicin was successfully delivered into the cancer cells, where it entered the nucleus to exert its cytotoxic effects [78]. It must be considered that in some of the works in the literature, in addition to loading a therapeutic cargo, modifications are made to the PDNPs that can improve or make the targeted administration more specific; that can increase their stability and favor homing to the tissue/organ of interest. These characteristics are mainly brought about by surface modifications that include coating the membrane with various substances. At the beginning of this section, work was already mentioned in which extracellular vesicles derived from lemon and loaded with doxorubicin were modified with heparin-cRGD (HRED). This functional modification resulted in a more stable nano-drug, as this coating was shown to have good anti-complement activation by inhibiting the formation of C3a to escape phagocytosis [68]. Another example of functional modification was realized by Zhang et al. in work conducted on mouse models of hepatocarcinoma with exosome-like nanovesicles derived from Asparagus cochinchinensis (ACNVs) [79]. ACNVs were engineered by incorporating DSPE- PEGs into lipid membranes to obtain PEG- ACNVs. This modification significantly improved the blood circulation period and enhanced the tumor-targeting capacity of ACNVs by increasing accumulation at tumor sites. In vivo pharmacodynamic studies showed that PEGylated ACNVs can better inhibit tumor growth without significant side effects [79]. And finally, Niu et al. created a biomimetic system from grapefruit extracellular vesicles by combining them with doxorubicin (DOX)-loaded heparin-based nanoparticles for highly efficient drug delivery in glioma treatment [80]. This system exploits in parallel the natural abilities of grapefruit EVs to efficiently cross physiological barriers such as the blood–brain barrier as well as the ability of heparin to increase the stability and improve the bioavailability of EVs in vivo, due to its property of escaping the immune system [80].

In Table 1 we reported the studies discussed in this section on the different loading strategies of PDNPs.

### 3.3. Nanovectors Made of PDNP Lipids

The field of drug delivery is constantly growing, as well as the need to find new systems to deliver a multitude of substances directly to the target site. In this regard, nanotechnology has made great contributions to the development of smart carriers in recent years. Among these, the possibility of engineering nanovectors from plant-derived extracellular vesicle lipids has emerged. Nanoparticles produced by PDNP lipid reassembly have innumerable advantages over synthetic carriers. Indeed, they have proven to be non-toxic and low-immunogenic, can be easily modified to make targeting specific, can be loaded with various substances while protecting them from the external environment, and can also be economically produced on a large scale in an eco-friendly manner [62,81,82]. The possibility of using PDNP lipids circumvents some of the problems of the use of EVs in drug delivery vehicles; among those, the exclusion of endogenous PDNP content, whose presence may interfere with the biological function of the system. Furthermore, the utilisation of the extrusion method offers the possibility of generating nanoparticles all of the same size [62,81,82]. The most widely used technique for obtaining nanovectors from PDNP lipids originates from the standard method of liposome assembly and is based on the two-phase liquid–liquid extraction method (LLE). It also exploits the natural propensity of amphipathic molecules such as phospholipids, of which PDNPs are mainly composed, to reassemble. For instance, Zhang et al. (2017) and Wang et al. (2019) produced lipid nanocarriers of relatively uniform size from ginger nanoparticles using a well-known LLE method: Bligh and Dyer [83,84]. Using this, the authors extracted total lipids from ginger nanoparticles and subsequently passed the extracted lipids through a 200 nm liposome extruder [83,84,85]. Through the same method, another group, Wang et al., extracted the entire lipid portion from grapefruit nanoparticles [86]. These were then sonicated and subsequently passed through a high-pressure homogenizer, allowing the lipids to reassemble into a multi-layered flower-like structure of about 200 nm in size [81,87]. Similarly, Teng et al. extracted the lipid components from grapefruit nanoparticles. Subsequently, reassembly into nanocarriers was carried out after UV irradiation in a Spectrolinker and bath sonication [88]. At the same time as lipid nanocarriers are created, they can be loaded with different types of cargo. In fact, the sonication step in the above-mentioned work is aimed at encapsulating the cargo within the reassembled nanoparticle. Furthermore, the nanocarriers can be decorated with molecules that improve delivery or uptake. In this regard, Wang et al., after developing grapefruit EV-derived nanocarriers (GNVs), demonstrated in a first study [81] their versatility as a transport carrier, being able to be efficiently internalized by various healthy or tumor cell types, both human and murine, without inducing cytotoxicity or affecting cell viability in any way. They also described their ability to transport various substances, including drugs, such as pentoxifylline to the target site and demonstrated their ability to inhibit tumor growth in vivo in two mouse models of tumor xenografts [81]. In a second study [86], they coated grapefruit-derived lipid nanocarriers with membranes enriched with inflammatory receptors of activated leucocytes (IGNVs). This modification greatly improved the homing of IGNVs to inflamed tissues and increased their ability to transmigrate through endothelial cells, exploiting the leukocyte-activated pathway led by factors related to leukocyte recruitment, including chemokines, chemokine receptors, and integrin. After establishing these characteristics, in the same study, the authors loaded the lipid nanovectors in question with drugs including chemotherapeutics such as doxorubicin, and anti-inflammatory agents such as curcumin. In this way, the therapeutic potential of IGNVs was further demonstrated, as they could enhance the therapeutic effect of chemotherapeutics by inducing a significant inhibition of tumor growth in vivo in two tumor models. They also enhanced the anti-inflammatory power of curcumin in mice with DSS-induced colitis [86]. Another example of how modifications to grapefruit nanovesicles (GNVs) can increase their specificity was demonstrated in a study by Tang et al. in which nanovesicles were conjugated with an aptamer (HA1) specifically targeting HER2+ breast cancer cells and loaded with doxorubicin, a well-known anticancer drug. The results showed that HA1 significantly enhanced GNVs’ internalization in MDA-MB-453 tumor cells and tumor spheroids and promoted targeted drug release leading to tumor growth inhibition in vitro with the highest efficacy compared to free Dox and GNVs-Dox. The data observed in vitro were also confirmed in vivo in MDA-MB-453 tumor-bearing SCID mice [89].

In another study, Teng et al. further explored the power of GNVs as mir-18 transporters for the treatment of metastatic liver tumors. In particular, they demonstrated that miR-18a, a known anti-tumor miRNA, encapsulated in GNVs mediates the inhibition of liver metastasis by inducing M1 macrophages, and by inhibiting M2 macrophages in the liver of metastatic colon cancer-bearing mice [88].

Nanovectors composed of lipids derived from PDNPs were also described by Zhang et al. [85]. The research group extracted nanoparticles from ginger and reassembled their lipids into ginger-derived nanovectors. They showed that the nanovesicles were efficiently taken up by colon cancer cells (Colon-26 cells), showing excellent biocompatibility up to 200 μmol/L. In contrast, cationic liposomes at the same concentrations reduced cell growth and increased the rate of apoptosis [85]. In this study, ginger-derived nanovectors loaded with doxorubicin and modified with a folic acid coating not only showed a better pH-dependent drug release profile than commercially available Dox-liposomes; they also successfully inhibited tumor growth in a Colon-26 tumor xenograft model when compared to the free drug [85]. In another paper, again Zhang et al. used the same nanovectors to treat ulcerative colitis. Nanovesicles were loaded with siRNA against CD98 (siRNA-CD98), a protein expressed in the small and large intestines and whose increased expression in the colon plays an important role in colitis and colitis-associated cancer. The results indeed indicate that an orally administered nanovector, containing the siRNA, was effectively targeted specifically to colon tissues, resulting in a reduction of CD98 expression even using a very low dose of the siRNA [83]. Ginger-derived nanovectors were also used by Wang et al. as siRNA transporters against the divalent metal ion transporter (Dmt1) in an attempt to demonstrate that inhibiting the expression of Dmt1 attenuated iron loading in a mouse model of hereditary hemochromatosis (HH). First, a nanovector containing Dmt1 siRNA was developed and tested in vitro on HEK293 and Colon-26 cells; subsequently, the siRNA-nanovector complex was coated with folic acid (FA) to specifically target the proximal small intestine. Finally, authors demonstrated that the oral administration of FA-nanovector, carrying Dmt1-siRNA, successfully blocked iron accumulation in the tissues of Hepc−/− mice without inducing any inflammation or toxicity [84].

An important characteristic of a drug vehicle is the ability to overcome biological barriers. Lipid-derived PDEV nanovectors seem to be good candidates. In a paper by Zhuang et al. (2016), the authors demonstrated that folic acid-coated nanovectors from grapefruit (FA-GNVs) effectively intranasally delivered miR-17 across the blood–brain barrier, leading to a delay in brain tumor growth [87].

**Figure 1:**
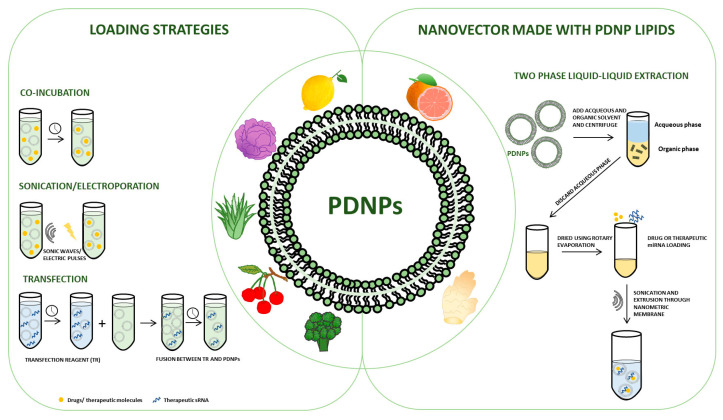
Schematic representation of the techniques used to date for: (left panel) the loading of exogenous substances into PDNPs; (right panel) the reassembly of lipids derived from PDNPs for the generation of natural nanovectors for use in the field of drug delivery.

Figure 1 represents a schematic representation of the already described studies on the use of PDNPs as drug delivery vehicles.

## 4. Conclusions

Nowadays there is an increasing urgency to find new systems for targeted and effective therapies. Therefore, in recent years, the scientific community has seen an exponential increase in studies aimed at finding new therapeutic answers with these characteristics for a wide variety of diseases. However, it is not easy to combine efficacy with the bioavailability of a drug. Therefore, new methods must be found to improve pharmacokinetics and clinical outcome. In this regard, extracellular vesicles, and in particular those derived from the plant kingdom, could be ideal candidates. They are natural transporters, involved in cell–cell communication mechanisms and created by nature to carry endogenous substances. In addition to their natural origin and the characteristics of safety, non-toxicity and low immunogenicity discussed at length in the previous sections, the possibility of isolating them from large volumes, and producing them on a large scale, also makes them very attractive for these purposes. Many research groups have ventured into engineering them, loading them with therapeutic substances, drugs and small therapeutic RNAs, and making functional surface modifications that increase their specificity towards a tissue or enhance their stability in circulation. Although the knowledge gained so far is an excellent starting point, we are only at the beginning and much still needs to be explored.

Indeed, despite the growing number of studies in this field and despite the promising premises, some open questions remain. Among these, the main challenges include the study of the origin of PDNPs; in this regard, it is important to emphasize that the biogenesis pathways are still unclear. Additionally, standardized procedures for PDNP isolation, characterization, and loading represent urgent needs.

We believe that through a multidisciplinary approach involving biologists, biophysics, clinicians, engineers, and chemists, much can be done to overcome these challenges and to translate current research into therapeutic opportunities.

Overall, the studies discussed in this review, albeit preliminary, are extremely encouraging, as they show how the synergy between the intrinsic therapeutic action of PDNPs combined with the possibility of carrying exogenous substances can lead to the development of innovative biological drug delivery systems, broadening the horizons of drug delivery through natural nanoplatforms.

## Figures and Tables

**Table 1:** Summary of the strategies for the exogenous loading of PDNPs.

Loading Approaches	Nanoparticles Sources	Cargos	References
Co-incubation	Aloe gel (gADNVs)	Indocyanine green (ICG)	[67]
Co-incubation	Lemon	HREDs Doxorubicin	[68]
Co-incubation	Grapefruit	methotrexate (MTX)	[59]
Incubation	Acerola	miRNAs(miR-340)	[69]
Sonication	Grapefruit	Anti-apoptotic protein (HSP-70)	[75]
Sonication	Bitter melon	5-fluorouracil (5-FU)	[76]
Transfection	Broccoli	miRNAs	[77]
Transfection and incubation	Cabbage/Red cabbage	miRNAs(miR-184)-doxorubicin	[78]

## Data Availability

No original data were generated in this review article.
